# Type 2 diabetes in East Asians: similarities and differences with populations in Europe and the United States

**DOI:** 10.1111/nyas.12098

**Published:** 2013-04-01

**Authors:** Ronald CW Ma, Juliana CN Chan

**Affiliations:** Department of Medicine and Therapeutics, Hong Kong Institute of Diabetes and Obesity, Li Ka Shing Institute of Health Sciences, The Chinese University of Hong Kong, The Prince of Wales HospitalHong Kong, China

**Keywords:** type 2 diabetes, East Asians, Chinese, Japanese, Koreans, diabetic complications, pathophysiology, genetics, β cell function, visceral adiposity, gestational diabetes

## Abstract

There is an epidemic of diabetes in Asia. Type 2 diabetes develops in East Asian patients at a lower mean body mass index (BMI) compared with those of European descent. At any given BMI, East Asians have a greater amount of body fat and a tendency to visceral adiposity. In Asian patients, diabetes develops at a younger age and is characterized by early β cell dysfunction in the setting of insulin resistance, with many requiring early insulin treatment. The increasing proportion of young-onset and childhood type 2 diabetes is posing a particular threat, with these patients being at increased risk of developing diabetic complications. East Asian patients with type 2 diabetes have a higher risk of developing renal complications than Europeans and, with regard to cardiovascular complications, a predisposition for developing strokes. In addition to cardiovascular–renal disease, cancer is emerging as the other main cause of mortality. While more research is needed to explain these interethnic differences, urgent and concerted actions are needed to raise awareness, facilitate early diagnosis, and encourage preventive strategies to combat these growing disease burdens.

## Introduction

The prevalence of diabetes is increasing globally, but the situation is particularly alarming in Asia. In the latest edition of *Diabetes Atlas*, an estimated 366 million individuals are affected globally, of whom 36% live in the Western Pacific region, with a significant proportion from East Asia.^1^

While it is evident that, for the same BMI, Asian populations have a higher prevalence of diabetes than their European counterparts,^2^ there is a need to better understand the factors underlying these interethnic differences.

This review summarizes and discusses the epidemiology, pathophysiology, genetics, and socioeconomic factors that may contribute to these interethnic differences. In addition to drawing on epidemiological and clinical studies performed in Asian populations, we also made reference to lessons learned from Asian subjects living in the United States, Europe, or Australia, notably the Japanese Americans in Seattle who have provided invaluable insight into the pathogenesis of type 2 diabetes (T2D) in people of East Asian origin.

## Epidemiology of diabetes in East Asia

Epidemiological studies have documented consistent increases in the prevalence of diabetes across different countries or areas in Asia.^2^–^5^ For example, the prevalence of diabetes in China has increased dramatically from around 1% in 1980 to the most recent estimate of 9.7% from a nationwide survey,^6^ with the disease trends strongly associated with urbanization.^7^ In a systematic review of 22 studies on diabetes prevalence and associated factors in China from 2000 to 2010, diabetes prevalence increased from 2.6% to 9.7% during this decade, with increasing age, urban residence, positive family history, obesity, and hypertension being common associated risk factors.^7^

This pattern of increasing prevalence over the last 20 years is mirrored in many Asian countries, as shown by data on the prevalence of known and undiagnosed diabetes, as well as impaired glucose tolerance (IGT) in East Asia (Table 1).^1^ In addition to ethnic differences within Asian populations, urbanization provides another source of variation in prevalence of diabetes in different areas. A comparison of 35 epidemiological studies from Mainland China, Hong Kong, and Taiwan, noted that Chinese patients living in Hong Kong and Taiwan have a higher prevalence of diabetes than Mainland Chinese.^8^ The proportion of undiagnosed diabetes was lower in studies from Hong Kong and Taiwan (52.6%, CI: 49.8%, 55.5%) compared to Mainland China (68.6%, CI: 67.4%, 69.7%). This proportion of undiagnosed diabetes was found to be decreased to around 60% in a recent nationwide study in China.^6^ By comparison, in the United States, only 7 million of the 25.8 million people (27%) with diabetes were undiagnosed.^9^

**Table 1:** Comparison of prevalence of diabetes, age distribution, and proportion undiagnosed in East Asian countries compared to the United States and Europe

		Estimated	Proportion	Estimated		Mean
		number of	of DM	proportion		diabetes-related
	Diabetes	people	subjects aged	of DM cases	IGT	expenditure per
	prevalence in	affected in	20–39 in	undiagnosed	prevalence in	person with
Country	2011 (%)	2011	2011 (%)	in 2011 (%)	2011 (%)	DM (USD)
China	9.29	90,045,980	15.1	56.9	2.41^a^	194
Hong Kong	9.38	525,390	7.4	46.7	14.87	2,059
Macau	7.49	32,710	10.0	46.7	5.69	480
Taiwan	9.59	1,664,540	9.59	46.7	11.61	1,314
Mongolia	6.74	117,460	43.6	56.9	7.86	107
Japan	11.2	10,674,320	5.9	46.7	14.3	3,266
Dem. People's Republic of Korea	9.08	1,507,500	13.1	63.0	10.83	17
Republic of Korea	8.8	3,186,390	10.0	46.7	13.45	1,615
USA	10.94	23,721,760	13.9	27.7	11.97	8,468
Canada	10.80	2,716,140	6.0	27.7	12.2	5,106
United Kingdom	6.84	3,063,910	6.95	36.6	9.19	4,267
Australia	8.10	1,292,090	8.46	46.7	9.94	4,878

Note: the data source is based on projections from epidemiological surveys. Data source: Diabetes Atlas, Fifth edition, 2011. International Diabetes Federation.^1^

a IGT prevalence figures reported in the above reference is markedly different to that reported in a recent nationwide study, which reported age-standardized prevalence of diabetes of 9.7%, and 15.5% for prediabetes, including 11.9% with IGT.

In addition to the large number of individuals with diagnosed and undiagnosed diabetes, there are many at-risk subjects with IGT in East Asian countries (Table 1). The contribution of IGT to the number of subjects with abnormal glucose regulation (IGT+DM) in Asian countries is high (Table 1). This ratio of IGT to total glucose intolerance, termed the epidemicity index, points to an even further increase in the future burden of diabetes in Asia.^10^

Globally, there is a general decrease in the age of diagnosis for diabetes,^11^ although the proportion of young- to middle-aged individuals with T2D remains higher in developing countries and Asia.^1^ In the Diabetes Epidemiology: Collaborative Analysis of Diagnostic Criteria in Asia (DECODA) study, which evaluated 19,845 subjects from Asia, it was noted that the age-specific prevalence of diabetes in urban Chinese and Japanese patients was higher than that in Europeans at 30–69 years of age from the Diabetes Epidemiology: Collaborative Analysis of Diagnostic criteria in Europe (DECODE) study.^12^,^13^ The prevalence and proportion of diabetes in patients aged 20–39 in different countries is compared in Table 1. In Taiwan, the prevalence of diagnosed diabetes increased substantially during 2000–2009, according to the Nationwide Health Insurance database. During this period, there was an 80% increase in the total number of diagnosed diabetic individuals, a 55% increase in the prevalence of diabetes, and a 35% increase in age-standardized prevalence. Overall, diabetes incidence increased by 25% during this period, with the increment in men higher than that in women. Alarmingly, in groups aged 20–39 and 40–59, the incidence and prevalence rates in men were 10% higher than those in women in 2000 and 40% higher in 2008.^14^

## Diagnosis of type 2 diabetes in East Asians

Traditionally, the American Diabetes Association (ADA) does not recommend the primary use of the 75 g oral glucose tolerance test (OGTT) to diagnose diabetes in routine clinical practice, although the OGTT remains the gold standard for diagnosis according to the World Health Organization (WHO).^15^,^16^ Compared with Europeans in the DECODE study, a higher proportion of Chinese and Japanese men and women display IGT, relative to isolated impaired fasting glucose, across most age groups.^12^,^13^ In several epidemiological surveys it was noted that omitting the OGTT and postprandial plasma glucose criteria for diagnosis of T2D would have led to significant underreporting of the prevalence of diabetes in Asian populations. For example, significant decreases in the prevalence of diabetes have resulted from the application of the 1997 ADA criteria compared with the WHO criteria in Hong Kong Chinese.^17^ Out of 1,513 individuals in a Hong Kong Chinese working population, 27 had a known history of diabetes. Out of the remaining 1486, 29 (1.95%), with a fasting glucose <7 mmol/L, had a 2-h plasma glucose ≥ 11.1 mmol/L, whereas 8 individuals (0.53%), without a prior diagnosis of diabetes according to the WHO criteria, had a fasting glucose between 7.0 and 7.8 mmol/L and were classified to have diabetes according to the 1997 ADA criteria.^17^ In the DECODA study, which involved 11 population-based studies from Asia, only 37% of subjects diagnosed with diabetes using either elevated fasting glucose or abnormal OGTT fulfilled both diagnostic criteria.^18^ As many as 44% of men and 50.1% of women diagnosed with diabetes in a recent epidemiological study in China had normal fasting, but isolated increased 2-h, plasma glucose.^6^ This highlights the importance of using OGTT, despite its inconvenience, to diagnose diabetes in Asian populations.

The ADA and WHO have both included levels of hemoglobin A1c (HbA_1c_) ≥ 6.5% as one of the diagnostic criteria for diabetes.^15^,^19^ This recommendation has negated the problem of day-to-day variability of plasma glucose values and the need for fasting and dietary preparations. However, HbA1c may be affected by a variety of genetic, hematological, or illness-related factors. For example, iron or vitamin B_12_ deficiency may lead to a falsely high HbA1c. Hemoglobinopathies may also affect HbA1c measurements, for example, due to altered amino acids on binding sites of immunoassays for HbA1c, or by causing variable interference in assays that utilize ion exchange chromatography.^19^ Importantly, there are significant ethnic disparities in the correlation between HbA1c and ambient blood glucose levels.^20^ This may be related to genetic differences in the concentration of hemoglobin, the rates of glycation, and either the life span or the number of red blood cells.^21^

In a study of 936 Canadians of South Asian, Chinese, or European descent, considerable ethnic variability was found in the sensitivity and specificity of HbA1c and fasting plasma glucose measurements to diagnose diabetes.^22^ A study involving 3,819 individuals with prediabetes from five ethnic groups in the United States noted that the mean (± SD) HbA1c values were 5.80% ± 0.44 among white individuals, 5.89% ± 0.46 among Hispanics, 5.96% ± 0.45 among Asians, 5.96% ± 0.46 among American Indians, and 6.19% ± 0.59 among black study participants.^23^ In a study conducted in Shanghai, China, an HbA1c threshold of 6.3% was found to be more appropriate and highly specific for detecting undiagnosed diabetes in Chinese adults.^24^ These variations in the selection of diagnostic tests and cutoff values can have significant clinical and public policy implications. Combining the sensitivity of fasting plasma glucose with the specificity of HbA1c enabled substantial increases in the rate of identifying prediabetes and diabetes in Asia using the 75 g OGTT as the reference test,^25^ the utility of which was confirmed in a multiethnic population.^22^

Over 10% of adults in China have prediabetes.^6^ The majority of Asian subjects with prediabetes have IGT as opposed to impaired fasting glucose (IFG), as was shown by the Shanghai Diabetes Study. Compared to subjects with normal glucose tolerance, subjects with IFG and IGT have a 15- and 9-fold increased risk of developing diabetes, respectively.^26^

## Pathophysiology of type 2 diabetes in East Asians

One of the most striking observations is the occurrence of diabetes in East Asian countries at a much lower mean BMI.^2^ While the prevalence of diabetes in the United States was 8.3% with a much higher population BMI, Asian populations had similar prevalence despite a much lower population BMI. Early studies in Hong Kong Chinese patients have highlighted that the risk of diabetes and other cardiometabolic risk factors started to increase at a BMI of around 23 kg/m^2^.^27^ This has subsequently been confirmed, and at each BMI category, Asians have greater adiposity than Caucasian subjects.^28^–^30^ On the basis of these observations, the Western Pacific Regional Office of the WHO, the International Association for the Study of Obesity (IASO), and the International Obesity Task Force (IOTF) defined overweight in Asians as having a BMI ≥ 23 kg/m^2^; obesity as BMI ≥ 25 kg/m^2^, and central obesity as BMI ≥ 90 cm in males and ≥ 80 cm in females.^31^ Despite ongoing debates regarding the appropriate BMI and waist circumference cut-off values for individual populations, it has been recognized by a WHO expert consultation that, among Asian populations, risk appears to increase from 22 to 25 kg/m^2^, while the cut-off point for high risk appears to range from 26 to 31 kg/m^2^. The consultation further recommended that, while the WHO BMI cut-off points of 18.5–24.9 kg/m^2^ (normal range), ≥25 kg/m^2^ (overweight), 25–29.9 kg/m^2^ (preobese), ≥ 30 kg/m^2^ (obesity), 30–39.9 kg/m^2^ (obese class I), 35–39.9 kg/m^2^ (obese class II), and ≥ 40 kg/m^2^ (obese class III) should be retained as international classification, the cut-off points of 23, 27.5, 32.5, and 37.5 kg/m^2^ should be considered points for public health action.^32^

The relationship between BMI and risk of diabetes in Asian populations was recently examined in a large cross-sectional pooled analysis of 900,000 individuals in the Asian Cohorts Consortium.^33^ There was a consistent association between BMI and diabetes in all cohorts. The relationship between general obesity and diabetes appears to be less steep in Asian populations, where obesity is associated with a 2.5- to 3-fold increased prevalence of diabetes, compared to 6- to 8-fold increases in risk observed in a U.S. population.^34^,^35^ The Obesity in Asia Collaboration also noted a weaker association between increasing BMI and diabetes in Asians compared with Caucasians, though this study pooled together different Asian ethnicities, including Chinese, Japanese, Koreans, Thai, and Asian Indians.^36^ This difference in the relationship between obesity and diabetes in Asians is partly because the risk of diabetes begins to increase at comparatively normal BMI in Asian populations, and overall obesity is not a good measure of actual cardiometabolic risk, as discussed in more detail below.

**Figure 1:**
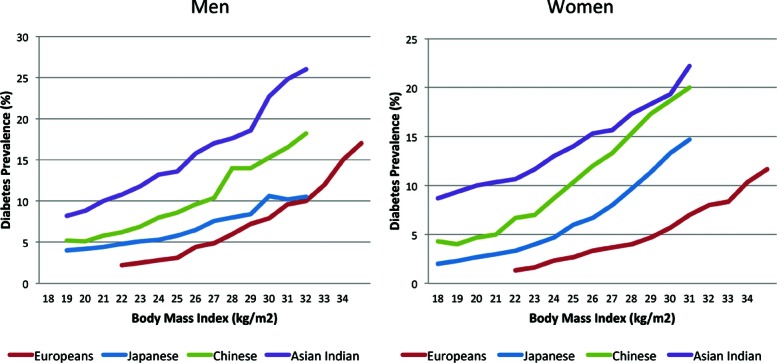
Relationship between BMI and diabetes prevalence in different ethnicities from the DECODA Study compared to a European population.^37^ Adapted with permission.

The DECODA study utilized cross-sectional data from 24,515 men and 29,952 women, and noted that the prevalence of diabetes was highest in Asian Indians, lowest in Europeans, and intermediate in Japanese and Chinese patients. The prevalence of undiagnosed diabetes increased with increasing BMI or waist circumference and to a similar extent in all ethnic groups, except Asian Indian women (Fig. 1).^37^

Given the inherent limitations of cross-sectional studies, heterogeneity in pooled analysis, and the large number of other factors associated with diabetes risks, studies that examined relationships between BMI and incident diabetes are more informative. A study that included data from the People's Republic of China Study and the Atherosclerosis Risk in Communities Study found larger associations between BMI and diabetes in Chinese Asians compared with American Whites.^38^ A multiethnic cohort of 59,824 subjects living in Ontario, Canada found that, for the equivalent incidence of diabetes at a BMI of 30 kg/m^2^ in white subjects, the BMI was 24 kg/m^2^ in South Asians and 25 kg/m^2^ in Chinese subjects. The study concluded that Chinese subjects developed diabetes at a higher rate, at an earlier age, and at lower ranges of BMI compared with Caucasians.^39^ In support of this increased risk of diabetes in East Asians, a recent 10-year multiethnic prospective study in the United States noted that, after adjusting for potential confounders, Asians had a hazard ratio of 1.86 for incident diabetes compared to 1.55 for Blacks and 1.67 for Hispanics (1.54–1.81), using a White population as the reference group.^40^

One possible reason for this interethnic difference is that, at any given BMI, compared to Caucasians, Asians have more visceral adiposity, which is metabolically more adverse, and contributes to lipotoxicity and insulin resistance.^29^,^41^ In comparative studies conducted in the United States, American Japanese patients have more visceral adiposity than their Caucasian counterparts.^42^

### Visceral adiposity

Central obesity, and in particular, visceral adiposity as measured by abdominal computed tomography or abdominal magnetic resonance imaging (MRI), is strongly associated with the risk of diabetes, independent of BMI, in both Europeans^35^,^43^ and Asians.^36^,^44^ In Japanese Americans, intraabdominal adiposity predicts subsequent development of diabetes, independent of age, gender, glucose tolerance status at baseline, family history, fasting C-peptide, and incremental insulin response.^45^ Increased visceral adiposity also increases the risk of IGT independent of insulin resistance, insulin secretion, and amount of other fat depots in Japanese Americans.^46^

Multiethnic studies have highlighted that, for any given BMI or waist circumference, Asians have greater adiposity or visceral fat than Caucasians.^29^,^41^,^47^ Generally, for the same BMI, body fat in Asians is higher by 3–5% compared with Caucasians.^41^ This tendency to visceral adiposity can lead to increased fatty acid influx to the liver, altered adipokine production, fatty liver, and hepatic insulin resistance.^48^ Nonalcoholic fatty liver, highly prevalent in Asia, predicts diabetes and cardiometabolic risk.^49^,^50^ Ectopic fat accumulation in the liver and skeletal muscle are important determinants of insulin resistance, which can propagate a vicious cycle with increased ectopic fat deposition in liver and islets, hepatic insulin resistance, β cell dysfunction and T2D.^51^

### Insulin resistance

While impaired β cell function and insulin resistance are both important in the pathogenesis of T2D, the relative contribution of these two factors in the etiology of diabetes varies in different populations.^52^,^53^ In a comparison of insulin sensitivity and β cell function in four different ethnic groups, Asian Americans were more insulin resistant than other ethnic groups, despite less obesity.^54^ As highlighted earlier, increased visceral adiposity in Asians may be one important factor. Visceral adiposity has been shown to predict changes in insulin resistance in a prospective cohort of Japanese Americans.^42^,^55^ This predisposition can be further exacerbated by lifestyle changes, as illustrated by increased obesity and insulin resistance in second generation Japanese Americans who have migrated to the United States compared to native Japanese.^42^ In a study of Japanese subjects with IGT from different age groups, young Japanese adults with IGT have the highest insulinogenic index, total insulin secretion, and homeostasis assessment of insulin resistance, highlighting that the marked insulin resistance present in these young subjects has already triggered the early manifestations of hyperglycemia.^56^,^57^

In addition to genetic and lifestyle factors, aging also increases the risk of visceral adiposity.^58^ In a study that compared insulin sensitivity and β cell function measured with a hyperglycaemic clamp versus estimated indices using formulas proposed by Matsuda and DeFronzo, or by Stumvoll in four ethnic groups, interethnic differences were detected only by clamp studies but not estimated indices.^59^ These findings have significant implications for the interpretation of current literature, as well as the design of future studies to examine ethnic differences in insulin sensitivity.

Adipose tissue is an active endocrine gland, and the distribution of body fat will affect adipokine production.^60^ Out of the different adipokines, adipocyte fatty acid binding protein (A-FABP) had the strongest association with insulin resistance, independent of adiposity, in Asian Americans of Chinese descent.^61^ In a 10-year prospective Chinese cohort, A-FABP was found to be an independent predictor of diabetes.^62^ Utilizing the metabolic syndrome as a proxy for insulin resistance, the presence of metabolic syndrome, defined by either National Cholesterol Education Program (NCEP) or International Diabetes Federation (IDF) criteria, was associated with a fourfold increased risk of developing diabetes over a median follow-up of 6.4 years.^63^

Mitochondrial dysfunction has been implicated in visceral adiposity, insulin resistance, and other metabolic abnormalities.^64^,^65^ Variants in mitochondrial DNA (mtDNA) may contribute to the pathophysiology of T2D. The mitochondrial DNA 3243 A to G mutation is a well-documented cause of diabetes in multiple ethnic groups and occurs in 3% of Asian patients with young-onset diabetes.^66^ The more common mtDNA 16189 variant has been reported to be associated with T2D in East Asians but not in European populations.^67^ Mitochondria may mediate changes in the epigenome to permit reversible modulation of gene expression in response to fluctuations in the energy environment. On the basis of this premise, it has been proposed that most DNA mutations may provide heritable and stable adaptation to regional differences in environmental factors.^68^

### Young-onset and childhood diabetes

Another feature characterizing T2D in Asian populations is the tendency to develop young-onset diabetes. Studies conducted in different East Asian populations have highlighted the earlier age at diagnosis. Among studies conducted in East Asia, the mean age of diagnosis of T2D is typically around 50 years. For example, in the Hong Kong Diabetes Registry, the mean age of diabetes diagnosis was 52 years.^69^ A multiethnic population-based cohort from Canada noted that the median age at diagnosis of diabetes was lower by 3 years in Chinese compared to Caucasians (55 years vs. 58 years), although the Chinese population from which cases were drawn was also slightly younger.^39^ Furthermore, the proportion of adults aged less than 40 years with a diagnosis of diabetes is higher in Asian countries compared to studies conducted in the United States and Europe (Table 1).^1^ For example, it is estimated that the proportion of diabetic individuals aged 20–39 years in the United States, Canada, and UK are 13.9%, 6%, and 6.9%, respectively. In contrast, the proportion of subjects with diabetes from the same age group in China, Taiwan, and South Korea are 15.1%, 9.59%, and 10%, respectively.^1^ In the 2005–2008 National Health and Nutrition Examination Survey (NHANES),^9^ the prevalence of diabetes among adults in the United States aged 20–44 years was 3.7%. In comparison, a survey of a nationally representative sample of 46,239 adults from China conducted between June 2007 and May 2008 showed a prevalence of diagnosed or undiagnosed diabetes among those aged 20–39 years to be 3.2%.^6^ Taking into consideration the more than 20-fold higher incidence of type 1 diabetes (T1D) in the United States and Europe compared to East Asian countries,^1^ this highlights possible differences in etiologies of young-onset diabetes in different geographical regions, with T1D being the more common cause of diabetes among adolescents and young adults in the United States and Europe, and T2D being a greater contributor to diabetes in East Asian populations. In line with this, T2D has already overtaken T1D as the more common form of childhood diabetes in Japan, Taiwan, and Hong Kong.^70^–^72^ By comparison, in the multiethnic SEARCH for Diabetes in Youth Study conducted between 2002 and 2003 in the United States among youths younger than 20 years, T2D remains relatively less common than T1D in Caucasians, and accounts for 14.9% of all diabetes cases among non-Hispanic white adolescents aged 10–20 years.^73^ The incidence rate of T2D was 5.6 per 100,000 person-years among non-Hispanic whites aged 15–19 years, rising to 22.7 per 100,000 person-years among Asians and Pacific Islanders in the same study.^73^

This increase in young-onset T2D is mainly due to the increasing prevalence of obesity, particularly among young men. In a study conducted in 2001 among 9,243 patients managed in 194 primary care centers across Asia, the proportion of T2D patients diagnosed before the age of 30 varied from 0.5% in Taiwan and 1.5% in Korea, to 5.4% in the Philippines.^74^ During the 1994–2000 period in China, there was an 88% increase in the prevalence of diabetes in a nationally representative population aged 35–44 years from the InterASIA study.^75^ A recent analysis of the National Health Insurance database from Taiwan noted that the prevalence of diagnosed diabetes among those aged 20–39 has increased by 15% (from 0.45 to 0.5) for women, and 54% (from 0.52 to 0.78) for men, during the 2000–2008 period.^14^

### Characteristics of young-onset diabetes

There is considerable heterogeneity among subjects with young-onset diabetes in Asia. A study of T2D patients in Hong Kong diagnosed before the age of 40 noted that 4% of patients had autoimmune markers, while half of them had a positive family history and/or obesity, suggesting that genetic factors and obesity play a greater pathogenetic role than autoimmunity.^66^ Another multicenter study of Asian patients diagnosed with diabetes before the age of 40 noted that only 11.5% were positive for autoantibodies against glutamic acid decarboxylase (GAD) or tyrosine phosphatase (IA-2A). Although the frequency of metabolic syndrome was lower in the group positive for autoantibodies versus those who were negative (27% vs. 54%), a substantial proportion of both groups of patients had atherogenic dyslipidemia, hypertension, and albuminuria. These studies highlight the phenotypic heterogeneity among young Asian patients with T2D. 3,76 In a more recent study of Asian Americans with young-onset diabetes, the difficulty in differentiating T1D from T2D based on clinical features was highlighted. Autoimmune markers were only present in 30% of patients with clinical presentation of T1D,^61^ similar to earlier findings in East Asian populations.^77^

Studies in different ethnic groups have reported an association of positive family history with a two- to sixfold increased risk of diabetes.^78^ In the nationwide survey conducted in China, the presence of a positive family history was associated with a threefold increase in the risk of diabetes.^6^ Similarly, a recent analysis from the NHANES III (conducted from 1988 to 1994) noted that the presence of a moderate family history (e.g., only one first-degree relative with diabetes) was associated with a 1.7-fold increased risk of undiagnosed diabetes.^79^ Familial diabetes is therefore an important risk factor in both East Asian and European populations. In the population-based study in China, up to 23% of newly diagnosed cases, and 43% of previously diagnosed cases, have a positive family history of diabetes.^6^

In a study comparing 377 Korean patients with positive family histories (mean age of diagnosis 47.2 ± 10.8 years) with 955 patients with negative family histories (mean age of onset 50.0 ± 11.2, *P* < 0.001), the former group had lower fasting c-peptide levels despite comparable plasma glucose levels. The association between positive family history and low c-peptide levels, consistent with impaired β cell function, remained significant after adjustment for age, gender, duration of diabetes, BMI, fasting plasma glucose, and free fatty acid levels.^81^ In another Korean study that examined the ratio of β cell to α cells in islets from donors, a positive relationship was found between BMI and β cell mass.^82^ In line with this observation, a consecutive cohort of Chinese patients with T2D found that those with low BMI had low fasting c-peptide levels, but high glycated hemoglobin levels.^83^

These studies suggest that, in Asian subjects with young-onset diabetes, genetically determined reduced β cell reserve contributes to an increased risk of diabetes in the setting of mild insulin resistance. 80,84 A significant proportion of patients with young onset diabetes who are not overweight have a positive family history of diabetes, presumably reflecting inheritance of genetic factors for T2D.^74^,^80^ Over recent years, it is increasingly appreciated that most of the common genetic variants for T2D impact on disease risk through impairment of β cell function.^4^,^85^–^88^ Among patients with young-onset diabetes in the Hong Kong Family Diabetes Study, there was significant familial aggregation of T2D and related phenotypes, including obesity, hypertension, and dyslipidemia, highlighting the interaction between genetic effects and shared environmental/lifestyle factors.^89^ Thus, positive family history, suggesting genetic and/or epigenetic factors, in combination with socioeconomic factors, may contribute to the early onset of disease in Asian populations (Figs. 3 and 5).

### β cell dysfunction

Impaired β cell function plays an important role in the pathogenesis of diabetes in Asians, especially in those who are not overweight or have a positive family history. Much of these insights came from pioneering work in the Japanese population. For example, reduced insulinogenic index, indicating reduced first-phase insulin secretion, was observed in overweight Japanese subjects who are normoglycemic. The failure to compensate for insulin resistance suggests that impaired insulin secretion is the predominant culprit in the pathogenesis of T2D.^90^ Similarly, in prediabetic Japanese with isolated IGT, reduced first-phase insulin secretion has been noted.^91^

While there appears to be a predisposition to impaired insulin secretion among East Asian populations, some studies have suggested ethnic differences within different East Asian populations. In a systematic review and meta-analysis, fasting serum insulin levels were found to be lower in Japanese than Chinese and Korean patients. Given the similar genetic background and body build among these ethnic groups, it was postulated that environmental factors such as different dietary habits might contribute to these differences.^92^

Several studies have compared the contribution of insulin secretion and resistance in the pathogenesis of diabetes in East Asians. For example, nonobese Japanese subjects with normal glucose tolerance, IGT, and T2D were evaluated using the minimal model approach.^93^ The subjects with IGT and diabetes displayed marked reduction in β cell function, indicated by reduced disposition index, but relatively little change in insulin sensitivity.^93^Similar findings were reported when using the estimated homeostatic model assessment (HOMA) index, highlighting the role of β cell dysfunction in Asians.^94^ The oral disposition index, indicated by the product of change in plasma insulin divided by change in plasma glucose during OGTT x insulin sensitivity, provides an estimate of β cell function relative to the prevailing insulin resistance. This index was found to be one of the best predictors for future development of T2D in Asian American and other populations.^95^,^96^ In a small study from Korea, the pancreatic β cell area in subjects who underwent pancreatectomies correlated with homeostasis model assessment of β cell function, the oral disposition index, and the c-peptide/glucose 30 min ratio.^97^

In a study that compared insulin sensitivity among nondiabetic premenopausal or early perimenopausal non-Hispanic white, African American, Chinese American, Japanese American, and non-Mexican American Latino women, women of East Asian origin (i.e., Japanese Americans and Chinese Americans) had lower HOMA-β than non-Hispanic whites after controlling for waist circumference, prevalence of IFG, triglycerides, and amount of alcohol consumed.^98^

### Role of amylin/amyloid deposits

Reduced amylin release was found to be a characteristic of T2D.^99^ Islet amyloid is derived from islet amyloid polypeptide (IAPP, amylin), which is coexpressed and cosecreted with insulin from pancreatic β cells. IAPP can form toxic oligomers, leading to β cell apoptosis, and is one of the important pathways contributing to β cell loss.^100^ Amyloid deposits in the pancreas have been reported in subjects with T2D of European or East Asian origins.^101^,^102^

### Autoimmunity

In the United Kingdom Prospective Diabetes Study (UKPDS), 12% of the 3,672 Caucasian patients with T2D had either autoantibodies to GAD or ICA, with another 4% being positive for both antibodies. Compared to older age groups, patients aged 25–34 had a higher frequency of autoantibodies, which may be as high as 21% for ICA and 34% for GAD.^103^ Although the frequency of autoantibodies against GAD has been reported at 2–5% in Asian patients with T2D,^80^,^104^,^105^ this may be even higher in young patients. In a selective group of patients with young-onset diabetes recruited for the Asian Young Diabetes (ASDIAB) Study, 11.5% of newly

diagnosed patients aged 12–40 were positive for autoantibodies to either GAD or IA-2A.^76^

Similar to what has been observed in Western populations,^106^ the prevalence of metabolic syndrome among Asian patients with late-onset autoimmune diabetes in adult (LADA) is less than that in T2D.^107^ In the ASDIAB Study, the prevalence of metabolic syndrome was higher among patients who were negative for autoantibodies than that in those who were positive (54% vs. 27%). In both groups, a substantial proportion had atherogenic dyslipidemia, hypertension, and albuminuria.^76^ Thus, although autoimmunity does not appear to be a common contributing factor toward β cell dysfunction among Asian patients with T2D, it may play a greater role among those with young-onset diabetes. These frequent, but not invariable, co-occurrences of autoimmunity, β cell dysfunction, and metabolic syndrome, contribute to the phenotypic heterogeneity in young Asian patients with diabetes.

### Genetic factors

Type 2 diabetes results from complex interactions between multiple genetic susceptibility factors, as well as environmental and behavioral factors. Recent advances have led to the discovery of more than 60 genetic variants associated with T2D.^85^,^88^ While genetic factors are often suspected to contribute to the ethnic disparities in relation to diabetes prevalence and clinical phenotype, most of the common genetic variants identified in T2D patients of European descent have also been replicated in East Asian populations.^4^,^87^,^108^,^109^ Substantial variations do exist in the genetic architecture in different populations, and therefore the precise location of risk alleles within a susceptibility region may vary in different ethnicities depending on the frequencies of the different alleles.

**Figure 2:**
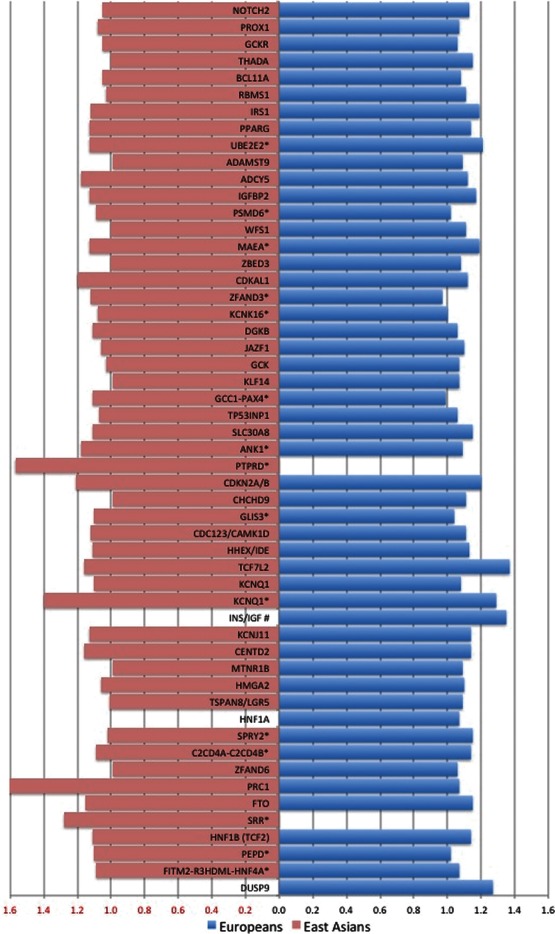
Comparison of effect sizes of type 2 diabetes risk in East Asians and Europeans for 53 confirmed single nucleotide polymorphisms. Effect size estimates, odds ratio (OR), were obtained from the East Asian genome-wide association (GWA) meta-analysis,^87^,^214^ whenever applicable, and from published meta-analyses in European populations.^86^,^88^ For a few loci, no corresponding data are available in other populations as yet. Locus marked with an asterisk (*) identifies the locus first identified in East Asian populations. Locus marked with # refers to a locus identified from an analysis including parent-of-origin effects.^215^

On the other hand, several T2D loci, including *KCNQ1*, were first discovered in East Asians and subsequently replicated in European populations. Given the higher frequency of the risk alleles in Asian populations, these loci may be particularly important in East Asian populations.^87^,^110^,^111^ A recent meta-analysis of East Asian studies has discovered eight novel loci for T2D, several of which showed suggestive associations in European populations.^87^ Most of these variants are predicted to influence the risk of T2D by affecting insulin secretion.^86^,^87^ The majority of loci reported to be associated with T2D show similar effect sizes in East Asian and European populations (Fig. 2).

**Figure 3:**
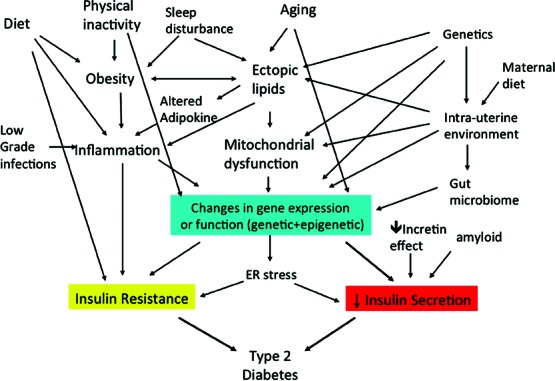
Pathogenesis of type 2 diabetes.

## Developmental origins and intrauterine environment

Aside from the inheritance of genetic factors, early life events and intrauterine environment can influence the development of β cell dysfunction and insulin resistance, which will affect the risk of obesity, T2D, and cardiovascular disease later in life.^112^,^113^ This is partly mediated by heritable alterations in gene expression that are not associated with changes in DNA sequence, but involve epigenetic modifications in the form of DNA methylation and histone modifications.^114^ Although the link between birthweight or maternal nutrition and adult risk of noncommunicable disease has been observed in populations of different ethnicities, it has been postulated that the impact is greatest among populations with the greatest mismatch between early life events and adult environment. Such a proposition may be most relevant to populations undergoing the most rapid socioeconomic development, such as Asia.^4^,^115^ In a Chinese study, offspring whose mothers were exposed to famine had increased risk of diabetes or hyperglycemia in adulthood. This risk was further increased if the offspring was exposed to a nutritionally rich urban environment.^116^

### Diabetes-complicating pregnancy and gestational diabetes

With earlier age at onset of diabetes, the proportion of pregnancies complicated by pre-existing diabetes is increasing in Asian countries^117^ and the United States^118^ In Pima Indians, exposure to intrauterine hyperglycemia was first reported to be associated with increased risk of diabetes and obesity in the offspring^119^,^120^ This effect has also been observed in the offspring of mothers with T1D, T2D, or gestational diabetes^121^,^122^ In addition to earlier age at diabetes onset,^123^ offspring exposed to intrauterine hyperglycemia have increased risk of end-stage renal disease.^124^

Gestational diabetes (GDM) is also increasing in prevalence, especially in Asian countries.^125^ For example, the adjusted prevalence of GDM has increased 2.8-fold between 1999 and 2008 in China.^126^ Increased risk of GDM among Asians occurs at a lower BMI. 50,127 Women with a history of GDM are at a sevenfold increased risk of diabetes later in life.^128^ Furthermore, offspring of mothers with GDM have increased adiposity at birth, and increased risk of diabetes and obesity later in life.^129^,^130^ A longitudinal cohort study of Chinese mothers with GDM found that their offspring (aged 8–10 years) who were exposed to intrauterine hyperglycemia have higher blood pressure, and more adverse cardiometabolic profile, than offspring of normoglycemic mothers.^131^ These offspring also have *in utero* hyperinsulinemia, as reflected by umbilical cord c-peptide concentration, which strongly predicts later risk of obesity and glucose intolerance.^132^ Given the potential transgenerational effects of maternal hyperglycemia, this vicious cycle of diabetes begetting diabetes is of particular concern in developing countries and Asia in driving the escalating epidemic of T2D.^117^

## The role of lifestyle and environmental risk factors

The changes in environmental factors in many East Asian countries mirror those seen in Western populations, but perhaps on a slightly different time scale. For example, there was a marked increase in fat intake among Japanese between 1945 and 1980, which has leveled out over the past two decades. Furthermore, there has also been a dramatic increase in the number of registered automobiles in Japan. These changes have been associated with a steady increase in BMI and cardiometabolic risk factors.^57^

A similar rise in cardiometabolic risk factors has been observed in many other Asian countries, which have undergone rapid economic development over the last few decades. A survey of nationally representative cohorts from the Asia-Pacific region noted that the prevalence of overweight and obese individuals has increased by 46% in Japan from 16.7% in 1976–1980 to 24% in 2000, and by 414% in China from 3.7% in 1982 to 19.0% in 2002, compared to a rise of 20% from the baseline prevalence rate of 50% in Australia over this time period.^133^ In China, metabolic syndrome has become increasingly common, and is estimated to contribute to 9 million deaths annually from cardiovascular diseases by 2030.^134^,^135^ Cancer and cardiovascular disease now account for approximately two-thirds of total deaths in the Chinese population aged 40 years or older.^136^

### Dietary factors

One of the major changes driving the diabetes and obesity epidemic is the rapid nutrition transition that is occurring in Asian countries.^137^ This includes a shift to higher fat and lower carbohydrate content in the diet, along with increased intake of foods from animal sources, edible oils, and added sugars. In China, the intake of dietary fat rose from 19.3% in 1989 to 27.3% in 1997.^138^ In China, the costs of diet-related noncommunicable diseases have already exceeded the costs of undernutrition.^137^

### White rice consumption

White rice is an important component of the daily diet in East Asians. Although the glycemic index of a specific white rice variety depends on a variety of factors, including the amount of processing, white rice in general has a higher glycemic index than whole grain rice.^139^ In Chinese and Japanese adults, there are positive associations between white rice consumption and risk of T2D.^140^,^141^ A recent meta-analysis and systematic review found that each increment in serving per day of white rice intake was associated with an 11% increase in the risk of T2D. This risk was significantly higher among Asian (Japanese and Chinese) than Western populations.^142^ These findings concur with earlier studies showing that Asian subjects had greater glycemic responses to the same foods compared to Caucasian subjects.^143^

### Smoking and environmental pollutants

Smoking is associated with increased risk of diabetes.^144^ This is of particular concern in some developing parts of Asia, where the rate of smoking is still increasing.^145^ Exposure to environmental organic pollutants such as bisphenol-A and organochlorine have also been found to be an important risk factor for diabetes and cardiometabolic risk.^146^

### Sleep-disordered breathing and sleep deprivation

The link between sleep disorders and T2D^147^ may be particularly relevant in Asia, where sleep time is comparatively shorter. For example, in a meta-analysis of data from 23 countries, it was noted that adolescents from Asia were sleeping 40–60 min less each night than Americans, and 60–120 min less each night than Europeans.^148^ Sleep-disordered breathing is associated with disrupted sleep and a twofold increase in the risk of diabetes, as well as poor glycemic control in diabetes patients.^149^ In Asia, an association between short sleep duration and increased risk of diabetes, childhood obesity, and cardiometabolic abnormalities has been reported.^150^–^153^

### The role of chronic infections

Several infections have been associated with increased risk of diabetes or related complications. Recent studies have reported an association between *Helicobacter pylori* and increased risk of diabetes,^154^ although other studies did not detect this association.^155^ Interestingly, studies conducted in East Asia where *H. pylori* infection is prevalent suggested a link between diabetes and gastric cancer.^156^ A recent study in Chinese patients noted an association between *H. pylori* titres and diabetes and postprandial blood glucose levels.^157^

Hepatitis B is endemic in Southeast Asia, and while not associated with increased risk of diabetes, chronic hepatitis B infection has been found to be associated with increased risk of GDM^158^ and end-stage renal disease among subjects with diabetes.^159^

### Multidimensional nature of diabetes

**Figure 4:**
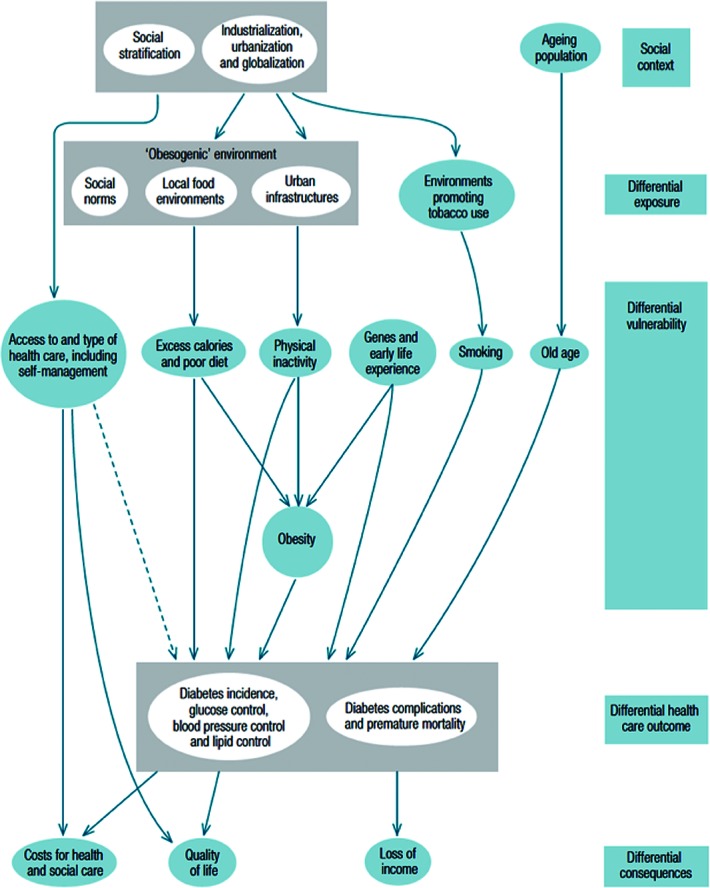
Social determinants of type 2 diabetes. Reproduced with permission from Whiting *et al*. 216

In many ways, diabetes is a biomedical consequence of rapid urbanization in genetically predisposed individuals. The rapid transitions from energy scarcity to energy abundance, physical activity to sedentary lifestyles, and a disease pattern dominated by infection to noncommunicable disease, are occurring in unprecedented and phenomenal rates in Asia. These factors can lead to biological, psychological, and behavioral maladaptation, possibly with a genetic or epigenetic basis. These (epi)gene–environment–lifestyle interactions are further influenced by socioeconomical factors, such as access to education, care, and support, resulting in a diversity of consequences (Fig. 4).

## Diabetic complications and comorbidities

In Asia, the increasingly young age of diabetes onset is likely to have significant implications for the pattern of complications and treatment strategies. Apart from genetic predisposition, young patients face long disease duration and greater tendency to β cell failure, which puts them at high risk for microvascular and macrovascular complications.

One of the earliest studies that compared the pattern of complications across different ethnicities was the WHO Multinational Study of Vascular Disease in Diabetes. In a subgroup analysis of this study, the incidence of vascular complications was compared among European, Asian, and American Indians patients with T1D or T2D who were diagnosed at less than 30 years of age. Among the Asian subjects, 62% were recruited from Tokyo and 38% from Hong Kong.^161^ This study highlighted the increased incidence of retinopathy, renal complications, and lower extremity amputations among American Indian men, and the higher incidence of ischemic heart disease among European women compared to the other cohorts. Asian diabetic men have a higher incidence of albuminuria compared to diabetic men in Europe.^161^,^162^ In another analysis that compared patients from the WHO Multinational Study of Vascular Disease in Diabetes with 447 Chinese patients with diabetes from Tianjin and Beijing, the prevalence of retinopathy and proteinuria were higher among Chinese patients, though the prevalence of large vessel disease was lower compared to the WHO cohort.^163^ An Australian study compared patients with T2D from six different ethnic groups, and noted Chinese and Indian subjects had a lower risk of hypertension, but a higher risk of albuminuria, compared to Anglo-Celtics.^164^

This pattern of increased microalbuminuria and renal complications in Asian patients with T2D has been observed across multiple studies, including other multiethnic studies. In the MicroAlbuminuria in Patients with Diabetes (MAP) study, as many as 58% of Asian patients with T2D and hypertension displaying evidence of renal injury, with 38% showing evidence of microalbuminuria, and a further 18% having macroalbuminuria.^165^ This is in contrast to population-based studies in Europe and the United States, which reported that the prevalence of microalbuminuria is between 17% and 21% among patients with diabetes.^166^ For example, in the UKPDS, the prevalence of microalbuminuria at the time of diagnosis of diabetes was 7.3%, increasing to 24.9% for microalbuminuria, and 5.3% for macroalbuminuria, 10 years after diagnosis of diabetes.^167^ In a cross-sectional study involving 32,208 patients with T2D from 33 countries (mean duration of diabetes was 7.6 years), the prevalence of microalbuminuria and macroalbuminuria was 39% and 10%, respectively. Asian patients had the highest prevalence of microalbuminuria and/or macroalbuminuria at 55% (43% for microalbuminuria, 12% for macroalbuminuria), compared to 40.6% in Caucasian subjects^168^ (33% for microalbuminuria, 7.6% for macroalbuminuria).

**Figure 5:**
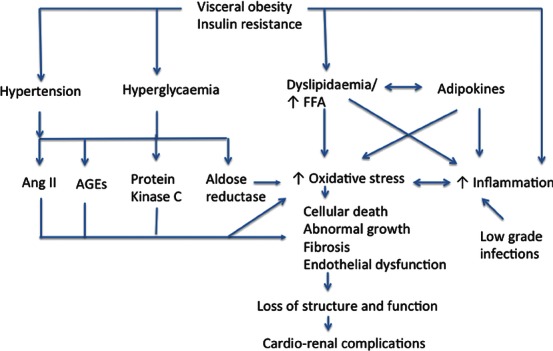
Pathogenetic pathways leading to diabetic cardio–renal complications. Adapted from Ref. 172.

In a study from New Zealand that examined the association between ethnicity and albuminuria among a large cohort of 65,171 subjects with T2D in a primary care setting, regression analysis revealed that, compared to subjects of European descent, the odds ratio of macroalbuminuria (defined as urinary albumin creatinine ratio (UACR) between 30 and 100 mg/mmol)) in East Asians was 2.9 (95% CI: 2.4–3.4), and advanced albuminuria (defined as UACR >100 mg/mmol) was 4.1 (95% CI: 3.2–5.1), after adjustment of risk factors, including age, gender, duration of diabetes, HbA1c, systolic blood pressure, triglyceride level, BMI, smoking, use of ACEI/ARB, and study site.^169^ This increase in risk of nephropathy in Asian patients is present even among nonhypertensive diabetic patients. Another multiethnic study of 2,969 primary care diabetic patients from a regional health maintenance organization in the United States noted that, among those patients without hypertension, the odds ratio of microalbuminuria was 2.0 in Asians compared to whites, and the odds ratio of macroalbuminuria was 3.2 in Asians, after adjustment of covariates, including ACEI/ARB use. Adjustment for BMI ≥ 30 kg/m^2^ and hypertriglyceridemia resulted in the greatest change in the odds of micro- or macroalbuminuria compared with whites, suggesting that obesity and dyslipidemia may play an important role in the pathogenesis of nephropathy in Asian populations (Fig. 5).^170^–^172^

Type 2 diabetes in Asian patients is characterized by an increased risk of renal complications but a lower rate of peripheral vascular disease (PVD) and amputations in a longitudinal multiethnic cohort from the Kaiser Permanente Medical Care Program in Northern California, which included 62,432 diabetic patients, 12% of which were Asians.^173^ In the Action in Diabetes and Vascular Disease (ADVANCE) study, patients with T2D from Asia had a higher incidence of renal complications and ischemic strokes, but lower risk of coronary heart disease (CHD) and PVD than their counterparts in Eastern Europe and other established economies such as Australia.^174^

Other studies supporting the lower incidence of CHD in Asian patients with T2D compared to Caucasians include the overestimation of CHD risk in Asian subjects by the UKPDS Risk Engine developed in Caucasian T2D patients.^175^ In the Asia Pacific Cohort Studies Collaboration, the leading cardiovascular cause of death was stroke (42%) in Asia, as opposed to CHD (59%) in Australia and New Zealand.^176^ Furthermore, the UKPDS identified clinical predictors of CHD as LDL cholesterol, HDL cholesterol, HbA1c, systolic blood pressure, and smoking.^177^ In Chinese patients with T2D, the main predictors of CHD were age, male gender, smoking status, duration of diabetes, reduced eGFR, albuminuria, and non-HDL cholesterol.^178^

In a recent meta-analysis, albuminuria and renal dysfunction were found to be independent predictors for all-cause and cardiovascular mortality, after adjusting for confounders.^179^ The prognostic significance of albuminuria is, in part, due to its close association with endothelial dysfunction.^172^,^180^ Consistent with this hypothesis, erectile dysfunction, which is a clinical marker of endothelial dysfunction, predicts CHD in both Asian and European subjects with T2D.^181^,^182^

In a subanalysis of the Japan Diabetes Complications Study, non-HDL and total/HDL cholesterol were the best predictors of CHD among diabetic men, whereas triglyceride was the best predictor for women.^183^ These findings show that, in addition to differences in the prevalence of diabetic complications (Table 2), there are differences in risk factors that predispose individuals to developing these complications between Asian and European populations (Fig. 5).^172^

**Table 2:** Prevalence of DM complications in different East Asian populations and comparison with selected studies in U.S. or European populations

Complication in different ethnic groups	Place of study	Setting	Number of subjects	Prevalence of complications	Other observations	Reference
**Diabetic retinopathy**						
	Hong Kong (1990–1996)E. Asian	T2DM referred to tertiary hospital clinic	474 patients with newly diagnosed T2DM within 1 year of diagnosis	Any DM retinopathy 21.9%		217
	Taiwan (data from different periods before 2000).E. Asian	Cross-sectional, T2DM patients in different centers	527 patients	Any DM retinopathy 35.0% (background retinopathy 30%, preproliferative retinopathy 2.8%, proliferative 2.2%)	Long duration of diabetes and use of insulin associated with risk of retinopathy	218
	Beijing, China (1995–1999)E. Asian	Cross-sectional, first time attendees at a Beijing Hospital	2,131 patients with T2 DM	Any DM retinopathy 27.3%, proliferative retinopathy 7.8% (21% of newly diagnosed patients have DM retinopathy)	Female gender; long duration of disease associated with risk of retinopathy	219
	Singapore Malay Eye Study SingaporeE. Asian	Population-based cross-sectional study	3,261 participants, 757 with diabetes	Any DM retinopathy 35%; vision threatening retinopathy 9.0%; macular oedema 5.7%	Predictors of retinopathy: increasing duration of DM and HbA1c; risk higher in females compared with men	220
	Handan Eye StudyE. Asian	Population-based cross-sectional study in rural China	6,830 adults	Any DM retinopathy 43.1%; vision threatening retinopathy 6.3%; macular oedema 5.2%	Duration of disease strongly associated with presence of retinopathy	221
	Chungu Metabolic Disease Cohort Study (2005–2006)E. Asian	Population-based cross-sectional study in rural Korea	1,298 adults with T2D	Any DM retinopathy 18%; severe nonproliferative or proliferative retinopathy 5%	Predictors: disease duration, postprandial glucose, and HbA1c	222
	Joint Asia Diabetes Evaluation (JADE) registry (2007–2009)Asian	Electronic registry of patients from 7 Asian countries, including Korea, Thailand, Hong Kong, Singapore, and the Philippines	3,487 patients with T2D	Any DM retinopathy: 20.4%		223
	National Health and Nutritional Survey in the United States (2005–2008)	Population-based cross-sectional study	1,006 adults with diabetes	Diabetic retinopathy 28.5%; vision threatening retinopathy 4.4%	Predictors of retinopathy: male sex, higher HbA1c, increasing duration of DM, insulin use, and blood pressure; males at twofold increased risk	224
	Multiethnic Study of Atherosclerosis (MESA) (U.S.)	Healthy population free of CV disease	778 adults aged 45–85 with diabetes	Prevalence of any retinopathy 33.2% and macular edema 9%; revalence of retinopathy 25.7% among Chinese Americans (c.f. 24.8% whites)	Predictors of retinopathy: longer duration of DM, higher FG, use of OHA or insulin, higher WHR; ethnicity not a risk factor for retinopathy	225
**Diabetic nephropathy**						
	MicroAlbuminuria Prevalence (MAP) studyAsian	Multicenter cross-sectional cohort study from 103 centers across Asia	5,549 patients with type 2 diabetes and hypertension	Microalbuminuria prevalence 39.8%; macroalbuminuria 18.8%	Predictors of microalbuminria: age, BMI, SBP, and ethnic origin; predictors of macroalbuminuria: age, gender, ethnic origin, BMI, DM duration, presence of complications, use of diuretics, use of CCB, DBP, and SBP	165
	Diabetes Study of Northern California (DISTANCE) (1996–2006)U.S.	Cohort study of patients in the Kaiser Permanente Northern California Diabetes Registry	64,211 patients with diabetes (including 40,286 whites, 1823 Chinese, 951 Japanese)	Incident ESRD 5.7 per 1,000 person-years for Chinese, 7.7 per 1,000 person-years Japanese, 3.9 per 1,000 person-years for whites	Increased risk of end-stage renal disease in Asians compared to white individuals	226
	National Programme in the New Zealand Diabetes Cohort Study (2000–2006)New Zealand	Cohort study of patients in primary care clinics in NZ	72,529 subjects with T2 DM in primary care; median duration DM 5.1 years; includes 1941 East Asians and 33,650 of European origin	Microalbuminuria present in 31% of East Asians, 28% of Europeans.	Odds ratio of microalbuminuria in East Asians compared with Europeans was 1.7 (1.5–2.0); macoalbuminuria or greater was 2.9 (2.4–3.4); advanced albuminuria was 4.1 (3.2–5.1) after adjustment of known clinical risk factors	169
**Diabetic neuropathy**						
	Hong Kong (1990–1996)E. Asian	T2DM referred to tertiary hospital clinic	350 patients with newly diagnosed T2DM within 1 year of diagnosis	Neuropathy (increased vibration threshold on biothesiometer) 12.8%		217
	National Health and Nutrition Examination Survey (1999–2004)U.S.		1,062 adults aged ≥40 with self-reported diabetes	Peripheral neuropathy defined as one or more insensate sites on monofilament testing; more likely if longer duration of disease present in approximately 28%	Peripheral neuropathy inversely associated with dietary intake of linolenic acid	227
**Cardiovascular disease**						
	Chinese patients with T2DM attending hospital clinics in major cities in China (Shanghai, Chengdu, Beijing, and Guangzhou)E. Asian	Cross-sectional survey of hospital clinics in 4 major cities in China	1,524 out-patients with T2 DM	30.1% with cardiovascular disease (including angina, myocardial infarction and CHF)		228
	Different DM centers in TaiwanE. Asian	Cross-sectional survey		15.8% had ischemic heart disease	Cholesterol and HbA1c associated with CHD risk	218
	Asia Pacific Cohort Studies collaboration (data up to 2000)Asian	24 cohort studies from Asia, Australia, and New Zealand	61,214 individuals (58% from Asia), of whom 4,873 has known DM at baseline	Overall hazard ratio of fatal coronary heart disease associated with DM was 2.19	No difference in risk of CHD conferred by DM in Asian versus non-Asian populations	176
	Diabetes Study of Northern California (DISTANCE) (1996–2006) (U.S.)	Cohort study of patients in the Kaiser Permanente Northern California Diabetes Registry	64,211 patients with diabetes (including 40,286 whites, 1,823 Chinese, 951 Japanese)	Incident MI in Chinese 9.2 per 1,000 person-years; incident MI in Japanese 10.5 per 1,000 person-years compared with 17.1 per 1,000 person-years in whites	Incidence for CHF also higher for whites than for Chinese/Japanese	226

Apart from microvascular and macrovascular complications, diabetes is associated with multiple morbidities including increased risk of various cancers, depression, mental illnesses, sleep apnea, respiratory disease, pneumonia, infections, osteoporosis, falls, dementia, and other degenerative conditions.^184^ Most of these associations, including cancer, have been reported in East Asian populations (Table 3). A recent meta-analysis of 33 studies reported that the association between diabetes and cancer was stronger in Asians compared to other populations.^185^

**Table 3:** Association of diabetes and cancer in East Asian and non-Asian populations

	Place of			Definition	Prevalence of	Other	
Comorbidity	study	Setting	Numbers	of endpoint	comorbidities	observations	Reference
Cancer	Hong Kong, China E. Asian	Hospital based diabetes registry, compared with Hong Kong cancer registry	6,107 T2 DM without known cancer at baseline	All-cause cancer or cancer-related admissions	All-cause cancer increased by 30% in diabetic men and women when compared to general population	Both low and elevated LDL cholesterol were associated with increased cancer risk in T2 DM	229, 230
Cancer	Korea E. Asian	10-year prospective cohort of 1.3 million Koreans (64% men)	20,566 cancer deaths in men; 5,907 cancer deaths in women	Cancer deaths	Fasting glucose ≥ 7.8 mmol/L; associated with 29% increase risk of cancer deaths	DM association with cancer of pancreas, esophagus, liver; colorectal cancer and cervical cancer	231
Cancer	Japan E. Asian	Meta-analysis of 4 cohort studies and 1 case–control study	22,485 cancer cases among 250,479 subjects with diabetes	All-cause cancer and site-specifc cancers	DM associated with 25% increase in risk of cancer in men; borderline significant increased risk in women	Risk of hepatocellular carcinoma increased by 3.6-fold in DM, risk of endometrial cancer 3.4-fold	185
Cancer	Emerging risk factors collaboration; Europe/USA	Collaborative analysis; 58% of studies from Europe, 36% from North America	802,900 adults from 97 cohort studies	Cause-specific deaths	DM associated with 25% increase risk of death from cancer	Increased risk of liver, pancreas, ovarian, colorectal, lung, breast and bladder cancer.	184
Cancer	Meta-analysis of studies in Asians versus non-Asians	Meta-analysis of published	33 studies; 156,132 subjects for mortality analysis; 993,884 subjects for incidence analysis	All-cause cancers	Cancer mortality 3%; cancer incidence 8%; pooled adjusted cancer mortality higher in DM compared to nondiabetic RR 1.32 (CI 1.20–1.45)	All-cause cancer incidence RR 1.23(1.09–1.39) In Asians compared to 1.15 (0.94–1.43) for non-Asians; incidence in Asian men significantly higher than non-Asian men	185

In addition to increased risk of different infections, diabetic patients have increased risk of sepsis that can lead to metabolic decompensation, morbidities, and mortality from infections.^160^ Diabetes increases the risk of tuberculosis by approximately twofold, as well as the risk of treatment failure.^160^,^186^ To this end, the dual burden of diabetes and tuberculosis is causing particular alarm in some Asian countries, such as China and India.^187^

## Treatment perspectives

### Public awareness and patient education

Despite the marked increase in diabetes prevalence in Asia, recent studies have highlighted a lack of awareness in many Asian patients. In the INTERASIA Study, only 24% of those with diabetes were aware of their diagnosis, with a large proportion of them being undiagnosed.^188^ Among subjects who reported a prior diagnosis of diabetes, only 85% were being treated with medications or nonpharmacological interventions. Among those treated, only 35% had a fasting blood glucose of < 126 mg/dL (<7 mmol/L).^188^ In the International Diabetes Management Practice Study (IDMPS), it was noted that lack of obesity and education on how to self-titrate insulin doses to self-monitor blood glucose levels were region-specific determinants of suboptimal glucose control among Asian patients with T2D.^189^

### Challenges in managing young-onset diabetes

The increasing number of patients with young-onset diabetes calls for urgent actions to empower these young individuals to achieve good glycemic control early to avoid long-term complications. In Asia, subjects with early-onset diabetes (age of diagnosis < 30 years) are characterized by worse glycemic control and higher frequency of eye complications compared to subjects with late onset of disease.^74^

Emotional distress has significant and negative impacts on self-efficacy, treatment compliance, and glycemic control. To this end, there is increasing recognition regarding the frequent co-occurrence of negative emotions including depression, anxiety, and stress in patients with diabetes, which markedly increase the risk of noncompliance, mortality, morbidity, and hospitalization. While the nature of these associations require further elucidation, a multipronged approach, including psychological support and empowerment are needed to improve the clinical outcomes and quality of life of these high-risk individuals.^190^

### Treatment strategies

Most of the antidiabetic medications currently in use in the United States and Europe are available in Asia, with some differences in drug formulations and dosing regimens. Although clinicians in Asia often refer to treatment guidelines developed by the ADA, European Association for the Study of Diabetes,^191^ the American Association of Clinical Endocrinologists (AACE),^192^ and the IDF,^193^ most countries in Asia have specific treatment algorithms or position statements developed by the local national diabetes organizations, in conjunction with local experts. While it is beyond the scope of this review to compare various national guidelines in Asia, the differences in prescription patterns may provide some insight into possible pathophysiological differences between T2D in East Asian, and European and U.S. populations.

One such example refers to α-glucosidase inhibitors (AGI). While acarbose is the only AGI available in most countries, there are three different AGIs (acarbose, voglibose, and miglitol) in Japan. In contrast to western countries where AGIs are used infrequently, acarbose is a popular treatment choice for both diabetes and IGT in China. This class of drug appears to be well tolerated and effective in East Asians, including Chinese and Japanese who tend to have high post-prandial glucose excursion, probably due to a combination of β cell insufficiency and high carbohydrate intake.^194^

Most pharmacoepidemiology studies in Asia date from the early 2000s, which reflect the status of diabetes treatment and drug choices at that time. In a survey conducted from 2000 to 2002 of over 16,000 Japanese diabetes patients registered in clinics and hospitals in the Japan Diabetes Clinical Data Management Study Group (JDDM), half of the patients were treated with oral antidiabetic drugs (OAD), one quarter were on either insulin or insulin plus OADs, and one quarter were treated by diet alone. Among the OADs prescribed, sulphonylureas were the most common drug class.^195^

In a hospital clinic-based cohort of 7,549 Chinese T2D subjects with a mean age of 57 years, mean disease duration of 6 years, and mean HbA1c of 7.7%, 7.9% of patients were on diet alone, 52.8% were on OADs, 6.5% were on insulin alone, and 32.7% were treated with a combination of OADs and insulin.^196^ Long disease duration was associated with more complex regimes in that combination therapy of OAD and insulin was prescribed in 23.7%, 39.3%, 57.1%, and 75.9% in those with disease duration of < 5 years, 5–9.9 years, 10–19.9 years, and ≥20 years respectively.^196^

In the National Health Interview Survey conducted from 2007 to 2009 in the United States among adults with diagnosed with T1D or T2D, 16% were taking neither OAD nor insulin, 58% only took OADs, 14% were on both insulin and OADs, and 12% were only on insulin.^9^ In a study of 5,135 T2D patients in Germany, monotherapy was the most common treatment regimen (43.3%), followed by a combination of OADs (10.9%) and OAD plus insulin treatment (4.4%). In Germany, monotherapy with metformin was more common than monotherapy with sulphonylureas (20.4% vs. 11.7%).^197^

In Asia, there has been much interest in the use of early insulin treatment. In newly diagnosed patients, intensive insulin therapy using continuous subcutaneous insulin infusion (CSII) improved β cell function, and in particular, first-phase insulin secretion.^198^ In a subsequent multicenter randomized clinical trial that recruited 382 newly diagnosed T2D patients, 2–4 weeks of intensive insulin treatment using CSII or multiple daily insulin (MDI) injections improved β cell function and induced a higher rate of disease remission (51.1% for CSII and 44.9% for MDI) at 1 year compared to use of sulphonylureas (26.7%, *P* = 0.0012).^199^ For the newer agents, such as the DPP-IV inhibitors and GLP-1 analogs, several studies have reported comparable or superior efficacy in Asian patients compared to Caucasian populations.^200^–^204^

While these studies are not directly comparable, the relatively high utilization of insulin secretagogues and insulin, as well as favorable responses to AGI and incretin-based therapy in Asian patients, support the importance of β cell dysfunction in these populations. On the other hand, glitazones, such as pioglitazone, are also prescribed in Asian populations, albeit at lower doses of 15–30 mg/day. This drug class has a particular place in therapy for subjects with fatty liver and metabolic syndrome, which are also prevalent in Asian subjects and may relieve β cell stress by alleviating insulin resistance. Given the double burden of β cell dysfunction and insulin resistance in Asian T2D patients, mediated in part by adiposity, there are strong arguments to advocate the use of phenotypes to guide therapies targeted at the predominant biological defect to achieve glycemic control early and preserve β cell function by reducing gluco-lipotoxicity.

Given the burden of cardiorenal complications in Asian populations, multifactorial management to reduce the risk of nephropathy is very important in this population. In a post hoc analysis of the Reduction of End Points in Type 2 Diabetes With the Angiotensin II Antagonist Losartan (RENAAL) Study, which examined the renoprotective effects of angiotensin receptor blockers (ARB) in T2D patients with renal insufficiency, treatment with losartan had a greater effect size in Asian populations, with a relative risk reduction of 35% compared to 18% in the main study cohort.^205^

## Economic impact

The potential impact of long-term complications on healthcare costs associated with suboptimal diabetes management is well documented (Table 1). In a cross-sectional study across four cities in China, direct medical costs for managing patients with microvascular and macrovascular complications increased by twofold compared to those without any complication.^206^

## Structured care and empowerment

To address the multiple needs of Asian T2D patients with complex phenotypes and to reduce the long-term risk of complications, researchers have developed an innovative and structured framework to facilitate early detection, education, monitoring, and care delivery. Given the broad coverage of internet and mobile technology in Asia, internet-based glucose monitoring systems and other mobile phone–based technologies have been shown to be effective in facilitating doctor–patient communications, enhancing self-monitoring, and improving glycemic control.^207^–^209^

Quality improvement initiatives targeted at the healthcare system and self-management were found to be particularly effective in improving risk-factor control in a recent meta-analysis.^210^ Using a team-based approach augmented by a protocol with predefined processes and targets, Asian researchers have shown the marked benefits of structured care in reducing the incidence of renal endpoints and related death in patients with T2D.^211^ This concept has been extended to a web-based disease management program, which incorporates a comprehensive risk engine, care protocols, and decision support, to promote self-management and enable physicians to establish disease registry and implement structured care.^212^

## Future perspectives

Recent work has highlighted the escalating burden of T2D in Asian populations, as well as some of the characteristics of diabetes in Asians. While studies suggest an important role of β cell dysfunction in the pathogenesis of T2D in East Asian populations, more detailed comparative studies are needed to further elucidate the underlying cause and metabolic defects. The search for genetic factors in East Asian and European populations has identified more similarities than differences between the different populations, although these loci only explained 10% of the heritability. From an evolutionary perspective, if the thrifty gene hypothesis, which states that genetic traits conducive to survival during times of hardship may promote risk of diabetes in times of affluence, such genotype(s) remains elusive.

On the other hand, there is a growing consensus on the possible causal role of early development and intrauterine environment, especially in regions undergoing rapid socioeconomic transition, with the greatest mismatch between the predicted prenatal and adult environments. How these factors may have contributed to the rapidly increasing prevalence of diabetes warrants further study. The rising prevalence of maternal diabetes, gestational diabetes, and childhood obesity will continue to fuel the epidemic, especially young-onset diabetes. To this end, a life-course approach to the prevention and control of diabetes is urgently needed, including a particular focus on early life opportunities.^213^

From a treatment perspective, clinical trials need to incorporate analyses or specific studies for Asian and other populations in order to identify treatment strategies that are most effective, given the phenotypic heterogeneity and interethnic differences in underlying pathophysiology, cultural, and lifestyle factors, as well as the pattern of diabetic complications.
